# Soluble Fms-Like Tyrosine Kinase-1 Alters Cellular Metabolism and Mitochondrial Bioenergetics in Preeclampsia

**DOI:** 10.3389/fphys.2018.00083

**Published:** 2018-03-06

**Authors:** Lissette C. Sánchez-Aranguren, Cindy T. Espinosa-González, Laura M. González-Ortiz, Sandra M. Sanabria-Barrera, Carlos E. Riaño-Medina, Andrés F. Nuñez, Asif Ahmed, Jeannette Vasquez-Vivar, Marcos López

**Affiliations:** ^1^Traslational Biomedical Research Group, Fundación Cardiovascular de Colombia, Santander, Colombia; ^2^Graduate Program in Biomedical Sciences, Faculty of Health, Universidad del Valle, Cali, Colombia; ^3^Maternal-Fetal Medicine Unit, Fundación Cardiovascular de Colombia, Santander, Colombia; ^4^Maternal-Fetal Medicine Unit, Clínica Materno Infantil San Luis, Bucaramanga, Santander, Colombia; ^5^Aston Medical Research Institute, Aston Medical School, Aston University, Birmingham, United Kingdom; ^6^Department of Biological Sciences, King Abdulaziz University, Jeddah, Saudi Arabia; ^7^Redox Biology Program and Free Radical Research Center, Department of Biophysics, Medical College of Wisconsin, Milwaukee, WI, United States

**Keywords:** preeclampsia, sFlt-1, mitochondrial dysfunction, endothelial dysfunction, oxidative stress and metabolic perturbations, metabolism

## Abstract

Preeclampsia is a maternal hypertensive disorder that affects up to 1 out of 12 pregnancies worldwide. It is characterized by proteinuria, endothelial dysfunction, and elevated levels of the soluble form of the vascular endothelial growth factor receptor-1 (VEGFR-1, known as sFlt-1). sFlt-1 effects are mediated in part by decreasing VEGF signaling. The direct effects of sFlt-1 on cellular metabolism and bioenergetics in preeclampsia, have not been established. The goal of this study was to evaluate whether sFlt-1 causes mitochondrial dysfunction leading to disruption of normal functioning in endothelial and placental cells in preeclampsia. Endothelial cells (ECs) and first-trimester trophoblast (HTR-8/SVneo) were treated with serum from preeclamptic women rich in sFlt-1 or with the recombinant protein. sFlt-1, dose-dependently inhibited ECs respiration and acidification rates indicating a metabolic phenotype switch enhancing glycolytic flux. HTR-8/SVneo displayed a strong basal glycolytic metabolism, remaining less sensitive to sFlt-1-induced mitochondrial impairment. Moreover, results obtained in ECs exposed to serum from preeclamptic subjects demonstrated that increased sFlt-1 leads to metabolic perturbations accountable for mitochondrial dysfunction observed in preeclampsia. sFlt-1 exacerbated mitochondrial reactive oxygen species (ROS) formation and mitochondrial membrane potential dissipation in ECs and trophoblasts exposed to serum from preeclamptic women. Forcing oxidative metabolism by culturing cells in galactose media, further sensitized cells to sFlt-1. This approach let us establish that sFlt-1 targets mitochondrial function in ECs. Effects of sFlt-1 on HTR-8/SVneo cells metabolism were amplified in galactose, demonstrating that sFlt-1 only target cells that rely mainly on oxidative metabolism. Together, our results establish the early metabolic perturbations induced by sFlt-1 and the resulting endothelial and mitochondrial dysfunction in preeclampsia.

## Introduction

Preeclampsia (PE) is an often-fatal cardiovascular complication related to pregnancy, that affects almost 8% of all pregnancies worldwide. Globally, it is associated with approximately 80,000 maternal and over 500,000 infant deaths annually, impacting the lives of over 4 million women worldwide(Duley, 2009). PE is characterized by maternal endothelial dysfunction (ED) (Powe et al., 2011; Sánchez-Aranguren et al., 2014), hypertension (LaMarca et al., 2008) and proteinuria (Maynard and Karumanchi, 2011). Alterations in circulating anti-angiogenic factors levels, such as the soluble form of the vascular endothelial growth factor (VEGF) receptor-1 (sVEGF-R1), commonly known as sFlt-1, have been associated to the onset of PE (Maynard et al., 2003; Levine et al., 2004, 2006). Nonetheless, the pathophysiology of PE remains unknown (Kanasaki and Kalluri, 2009).

In normal pregnancies, uterine blood flow increases to allow adequate perfusion of the placental intervillous space and physiological oxidative stress (Chaiworapongsa et al., 2014). In PE, a prolonged hypoxic placental microenvironment, due to a reduction in placental perfusion and oxygen availability, results in exacerbated oxidative stress (Chaiworapongsa et al., 2014). Hypoxia triggers several cellular responses, including increased placental angiogenesis (Zamudio, 2003), cell survival and metabolic adaptations (Illsley et al., 1984), established in developmental biology as “placental metabolic reprogramming” (Illsley et al., 2010; Jose et al., 2011).

Mitochondrial activity is essential in pregnancy because it sustains the metabolic activity of the placenta throughout gestation (LaMarca et al., 2008). Recently, a potential association between increased soluble anti-angiogenic factors levels and mitochondrial dysfunction has been suggested (Jiang et al., 2015). Exogenous administration of sFlt-1 in pregnant mice have shown to induce placental mitochondrial swelling, oxidative stress and apoptosis in trophoblasts (Jiang et al., 2015). In addition, preeclamptic plasma mediators induced deleterious effects on mitochondrial function of human umbilical vein endothelial cells (HUVEC) (McCarthy and Kenny, 2016). Together, findings suggest that mitochondrial function plays an important role in the onset of PE. Nevertheless, the role of early dysregulated sFlt-1 levels in PE, to induce perturbations in the mitochondrial oxygen consumption and bioenergetics in endothelium and placenta, has not been established.

Here, we report the effects of PE serum on mitochondrial oxygen consumption and metabolism in ECs and first-trimester extravillous trophoblasts (HTR-8/SVneo). As early elevated circulating levels of sFlt-1 are known to be implicated in the development of the disease (Maynard et al., 2003; Levine et al., 2004, 2006), we also established the effects of increasing levels of exogenous sFlt-1 on mitochondrial function. We demonstrate that PE serum significantly affects mitochondrial maximal respiration and spare respiratory capacity of ECs and trophoblast, enhancing a metabolic glycolytic phenotype. In addition, PE serum-induced mitochondrial reactive oxygen species (mtROS) formation. These effects were partially abrogated by exogenous VEGF. Finally, sFlt-1 treatment caused a dose-dependent loss of mitochondrial oxygen consumption in ECs and trophoblasts, affecting mitochondrial maximal respirations and spare respiratory capacities, and, inducing a metabolic phenotype switch to glycolysis, only in ECs. Our results provide novel insights on the differential metabolic perturbations exerted by sFlt-1 in the endothelium and placenta and their overall role in the development of PE.

## Materials and methods

### Subjects

Antecubital blood samples were collected from preeclamptic (PE) (*n* = 23) and normotensive women (NOR) (*n* = 23), before cesarean delivery. Subjects were recruited from the Maternal Fetal Units of Fundación Cardiovascular de Colombia (FCV), Floridablanca, Colombia and Clínica Materno Infantil San Luis (CMISL), Bucaramanga, Colombia, using protocols approved by hospital respective Ethics Committees. Ten non-pregnant subjects were recruited as controls (CTL). Preeclampsia diagnosis was established according to the American College of Obstetricians and Gynecologists (American College Obstetricians Gynecologist Task Force on Hypertension in Pregnancy, 2013) criteria (Supplementary Table 1). Subjects gave informed consent for their inclusion in the study.

### Cell culture

Bovine aortic endothelial cells (ECs) (passages 5–8) were obtained from Cell Applications, Inc. (San Diego, CA, USA). ECs were cultured in DMEM supplemented with 10% FBS (CellGro), 5.5 mM glucose, 4 mM L-glutamine, 1 mM sodium pyruvate and 100 U/ml penicillin/streptomycin at 37°C and 5% CO_2_. Prior to experiments, ECs were cultured in DMEM containing 2% FBS for 24 h. Extravillous trophoblast cells (HTR-8/SVneo, passages 75–82), are a well characterized, authenticated and immortalized first-trimester extravillous trophoblast (EVT) cell line(Graham et al., 1993). HTR-8/SVneo were cultured in RPMI-1640 (Gibco, Grand Island, NY, USA) supplemented with 5% FBS and 100 U/ml penicillin/streptomycin at 37°C and 5% CO_2._ To force mitochondrial metabolism, ECs and HTR-8/SVneo cells were grown in galactose supplemented media, matching the same glucose concentrations used previously, for two passages prior the analysis. Human recombinant sFlt-1 (VEGF-R1/Flt-1), and VEGF were obtained from R&D Systems, (Minneapolis, MN, USA). For *in vitro* correlation studies, cells were exposed to 2% serum from recruited women (Rodgers et al., 1988; Maynard et al., 2003).

### Cell viability assays

MTT assay was performed as cell viability assays. Cells were seeded at a density of 1 × 10^4^ cells/well on 96-well plates and cultured for 24 h. Then, cells were treated with 50 ng/mL of sFlt-1 recombinant protein (Novoprotein Scientific, Summit, NJ) for another 24 h. Cells were cultured in glucose and galactose supplemented media. After treatments, cells were exposed to 100 μL of MTT solution (5 mg/mL). Two hours later, formazan crystals were solubilized in dimethyl sulfoxide (DMSO). Absorbance was measured in a plate reader at 570 nm using a Varioskan Flash multimodal plate reader (Thermo Fisher Scientific, Vantaa, Finland).

### Mitochondrial oxygen consumption

Mitochondrial bioenergetics was assessed using an XFe24 Extracellular Flux Analyzer (Agilent Seahorse, Billerica, MA, USA). ECs and HTR-8/SVneo were seeded in V7 Seahorse micro-well plates at 3.5–4.0 × 10^4^ cells/well in 100 μL standard growth media. Cells were treated with sFlt-1 recombinant protein (Novoprotein Scientific) and 2% serum from recruited women, respectively, and incubated at 37°C and 5% CO_2_ for 24 h. Following treatments, culture media was changed to a non-buffered DMEM media, to allow temperature and pH equilibrium. Initially, oxygen consumption rates (OCR) and extracellular acidification rates (ECAR) were measured simultaneously three times to establish a baseline rate. Then, to evaluate mitochondrial function, oligomycin (1 mM) (Sigma Aldrich), carbonyl cyanide 4-(trifluoromethoxy)phenylhydrazone (FCCP) (0.5 mM) (Sigma Aldrich) and a mixture of rotenone and antimycin A (Rot/AntA) (1 mM) (Cayman Chemicals) were injected into each well sequentially, with intervals of 3–5 min of mixing between the injections, to respectively inhibit the ATP synthase, uncouple oxidative phosphorylation, and estimate non-mitochondrial respiration. Cellular respiratory control ratio (RCR) was obtained as the ratio of basal and oligomycin-inhibited (basal RCR) and FCCP-stimulated and oligomycin-inhibited mitochondrial respiratory rates (maximal RCR) (Brand and Nicholls, 2011; Dranka et al., 2011). OCR and ECAR measurements were normalized to protein content by the Bradford method (Supplementary Methods and Supplementary Figure 1).

### Aerobic glycolysis

Treated cells were washed and subjected to glucose (5.5 mM) (Sigma Aldrich), oligomycin (1 mM) (Sigma Aldrich) and 2-deoxi-glucose (2-DG) (100 mM) (Sigma Aldrich), subsequently, to respectively induce glycolysis, inhibit ATP synthase and estimate non-glycolytic acidification. Measurements were normalized to protein content by the Bradford method (Supplementary Methods and Supplementary Figure 1).

### Mitochondrial ROS formation

Mitochondrial ROS production (mtROS) was evaluated by fluorescence microscopy, using the fluorescent probe MitoSOX Red (Invitrogen, Oregon, USA). Briefly, ECs and trophoblast cells were cultured in 24-well plates, exposed to serum from recruited women and exogenous sFlt-1 (50 ng/mL), respectively, for 24 h. Cells were washed twice with HBSS and incubated with 5 μM MitoSOX Red probe in HBSS for 15 min at 37°C in 5% CO_2_, protected from light. Then, cells were washed again with HBSS and the red fluorescence emitted at 580 nm was analyzed, using a Nikon Eclipse Ti-S inverted microscope and NiS Elements software.

### Mitochondrial membrane potential

Changes in mitochondrial membrane potential (ΔΨ*m*) were performed using the JC-1 fluorescent dye by fluorescence microscopy (Invitrogen, Oregon, USA). JC-1 accumulates within the mitochondria and forms aggregates that emit red-orange fluorescence (Ex: 550/Em: 600 nm). When the Ψ*m* is dissipated, cells emit green fluorescence (Ex: 485/Em: 535 nm). Briefly, ECs and HTR-8/SVneo cells were cultured in 24-well plates at densities of 5.0 × 10^4^ cells/well. Treatments with sFlt-1 or serum were applied for 24 h. Then, cells were washed twice with sterile Phosphate Buffered Saline (PBS) at 37°C, followed by incubation with 5 μM of JC-1 in culture media for 30 min at 37°C, protected from light. The red/green fluorescence was evaluated using a Nikon Eclipse Ti-S inverted microscope and NiS Elements software.

### Statistical analyses

Each test was performed in three independent experiments. Data were plotted as means ± SEM. Statistical analysis was performed using Student *T*-test and ANOVA with Bonferroni's *post hoc* test, using Stata 8 statistical software (StataCorp, TX, USA). Values of *p* < 0.05, *p* < 0.01, and *p* < 0.001 were considered statistically significant.

## Results

### *In vitro* correlation model of PE: role of VEGF in mitochondrial bioenergetics

Early metabolic perturbations are considered the hallmark of common human diseases (DeBerardinis and Thompson, 2012). To understand the metabolic basis that would accompany the onset of PE, we assessed the cellular bioenergetics profile of ECs and HTR-8/SVneo cells, exposed to serum from pregnant and not pregnant women. For these purposes, we established a case-control study in where pregnant preeclamptic (PE), normotensive (NOR), and non-pregnant women (CTL), were recruited (Supplementary Table 1). Circulating sFlt-1 levels were determined in recruited women, to corroborate preeclamptic conditions. As reported previously, sFlt-1 levels were increased in PE women (Maynard et al., 2003; Levine et al., 2004) by several orders of magnitude in comparison with NOR and CTL patients (Supplementary Figure 2). Then, serum from all groups was used to replace FBS at 2% in culture media, to establish an *in vitro* correlation model (Rodgers et al., 1988; Maynard et al., 2003) of PE. Next, the effects in mitochondrial metabolism and bioenergetics were assessed, using Agilent-Seahorse technology (Dranka et al., 2011) as described in methods sections.

**Figure 1:**
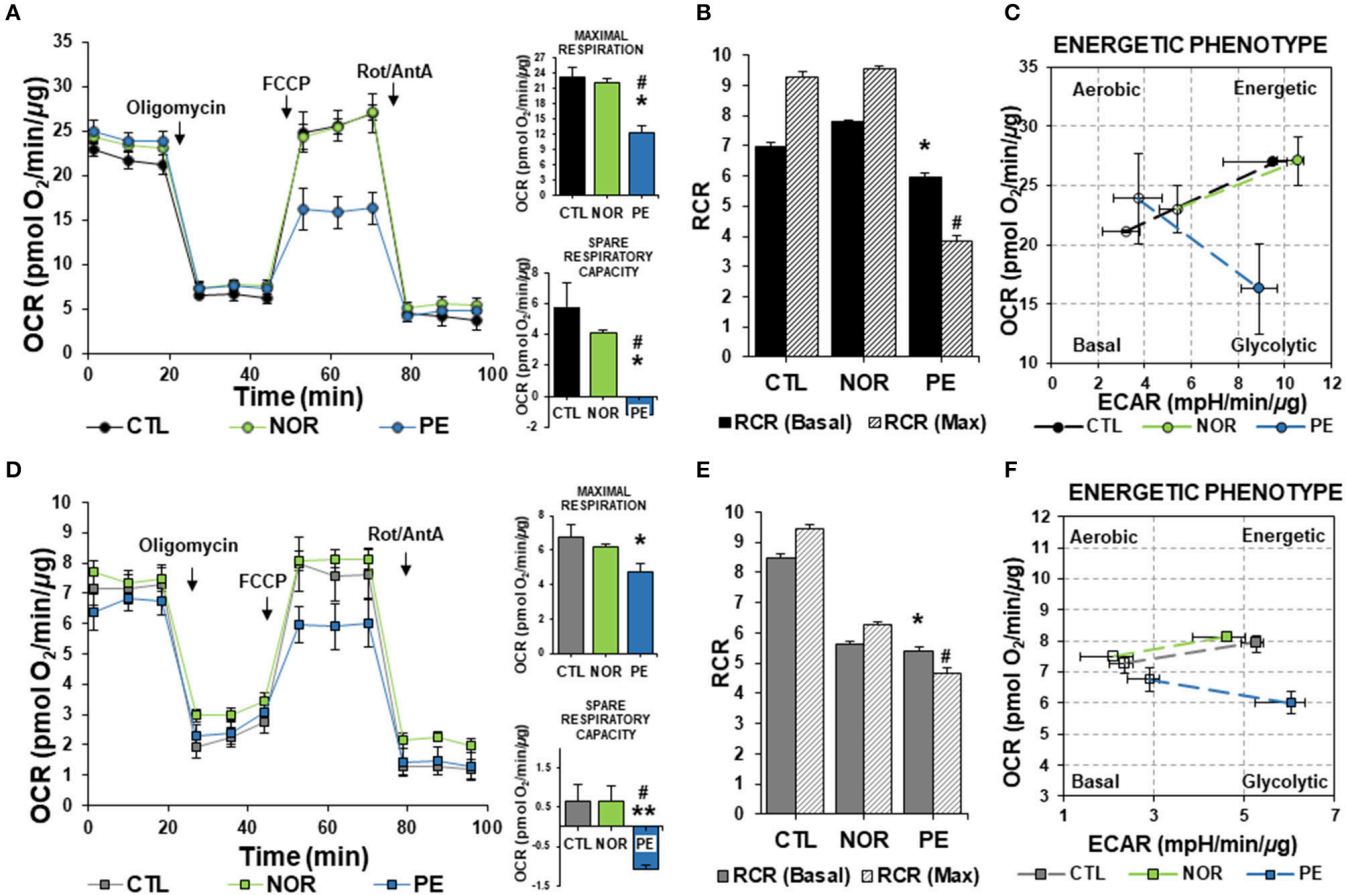
Bioenergetics profile of cells treated with serum from PE women. ECs and HTR-8/SVneo cells were treated with 2% serum from NOR, CTL, and PE women for 24 h. **(A)** Basal oxygen consumption rates (OCR), maximal respiration (OCR after FCCP administration) and spare respiratory capacity (Difference between basal and maximal OCR), **(B)** Ratios of basal and maximal (Max) respiratory control (RCR) (State 3/state 4), **(C)** Energy phenotype map of basal and maximal OCR and extracellular acidification rates (ECAR), measured in ECs. **(D–F)**, are the same experiments as in **(A–C)**, respectively, but evaluated in HTR-8/SVneo cells. Data are presented as means ± SEM. (*n* = 5). In **(A,D)**: ^*^P < 0.05, ^**^P < 0.01 vs. CTL, ^#^*P* < 0.05, vs. NOR. In **(B**,**E)**: ^*^P < 0.05, vs. basal CTL RCR, ^#^*P* < 0.05, vs. CTL maximal RCR. ANOVA (*Bonferroni's post-hoc test*).

As evidenced in Figure 1A respiratory traces show that treatment of ECs with serum from PE women induced a profound change in mitochondrial respiration rates. Maximal respiration and spare respiratory capacity rates were calculated after FCCP administration. FCCP induces the collapse of the mitochondrial membrane potential, leading to the determination of maximum OCR (Brand and Nicholls, 2011; Dranka et al., 2011). Administration of CTL and NOR serum did not affect respiration rates (Figure 1A). As observed in cell respiratory control ratios (RCR) (State 3/State 4), PE serum significantly affected mitochondrial respiration profile, suggesting mitochondrial dysfunction, associated with a low substrate oxidation capacity (Figure 1B).

Interestingly, when we assessed the cell energy phenotype of ECs, we found a slight increase in basal OCR in ECs treated with NOR and PE serum. However, when cells were challenged with FCCP to uncouple mitochondria, only CTL and NOR treated cells showed an increase in both OCR and ECAR. ECs treated with PE serum exhibited a drastic drop in OCR below basal levels, with a non-significant increase in glycolytic function (Figure 1C). This suggested that ECs under PE conditions (modeled with serum from women with PE), are not going to be able to cope with physiological challenges that will require an increase in energy utilization via mitochondria.

To establish the effects of PE serum in placenta, we used HTR-8/SVneo cells, which is an immortalized first trimester EVT cell line, as our model. Since early perturbations of sFlt-1 will be developing before the 20th-week of gestation, a trophoblast cell line that resembles the metabolic profile from those early stages was used. Results obtained after treatment with serum from PE women showed a reduction in maximal respiration in comparison with NOR and CTL serum of about 30% (Figure 1D). As in ECs, PE serum depleted the spare respiratory capacity rates. RCR determinations identified reduced oxidative phosphorylation in NOR serum-treated trophoblasts, along with an enhanced glycolytic response (Figure 1E). As observed in ECs, PE serum induced a significant decrease in OCR parameters, consistent with a weak coupling of respiration for ATP synthesis and enhanced ability to activate glycolytic pathways (Figures 1D–F).

Together, these results suggest that under full manifestation of PE conditions, the combination of several PE-key players, along with upregulated sFlt-1 (Maynard et al., 2003; Levine et al., 2004, 2006; Tosun et al., 2010; Vitoratos et al., 2010), will induce an insightful effect in the energetic phenotype of both, ECs and trophoblast, that will not allow them to metabolically respond to harsh conditions.

### Role of VEGF in mitochondrial function in PE

Once the impact of PE serum was established in ECs and trophoblasts, it was imperative to verify the implications of sFlt-1 in these observations. Although sFlt-1 levels in PE serum were higher than in NOR and CTL, other factors like pro-inflammatory molecules (i.e., TNF-α and IL-6), are known to be present in serum as described extensively in the literature (Tosun et al., 2010; Vitoratos et al., 2010), contributing with sFlt-1 to the effects observed.

**Figure 2:**
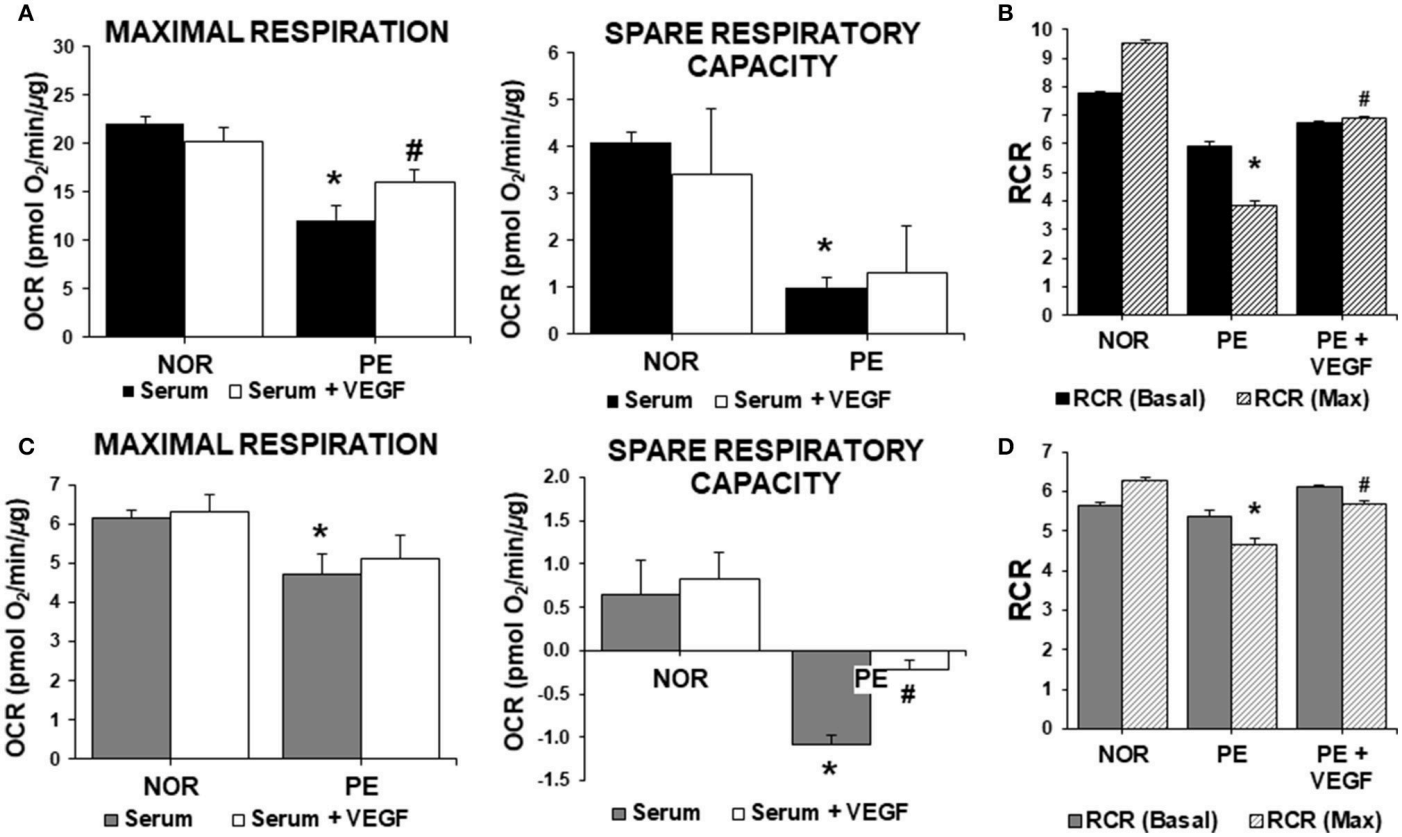
Role of VEGF in mitochondrial bioenergetics in preeclampsia. ECs and HTR-8/SVneo cells were treated with 2% serum from NOR, CTL, and PE women with and without exogenous VEGF (20 ng/mL) for 24 h. **(A)** Maximal respiration (OCR after FCCP administration) and spare respiratory capacity (Difference between basal and maximal OCR) in endothelial cells, **(B)** Basal and maximal (Max) RCR, **(C,D)** are the same experiments as in **(A,B)**, respectively, but evaluated in HTR-8/SVneo cells. Data are presented as means ± SEM. (*n* = 5). In **(A,C)**: ^*^P < 0.05, vs. NOR, ^#^*P* < 0.05, vs. cells exposed to PE serum alone. In **(B,D)**: ^*^P < 0.05, vs. NOR maximal RCR, ^#^*P* < 0.05, vs. PE maximal RCR. ANOVA (*Bonferroni's post hoc test*).

To address the role of sFlt-1 and VEGF bioavailabilities in the early metabolic perturbations in PE, VEGF (20 ng/mL) was administered to cells exposed to serum. VEGF administration should discriminate the antagonizing effects of sFlt-1 in serum from other possible factors. ECs and trophoblasts were treated with serum alone, and serum containing VEGF. As shown in (Figure 2A), VEGF administration to ECs treated with NOR serum did not affect maximal respiration or spare respiratory capacity. However, PE serum impaired the maximal respiration and spare respiratory capacity of ECs (Figure 1A) and these effects were partially reverted by VEGF (Figure 2A). This suggests that reduced VEGF bioavailability, due to sFlt-1 up-regulation, affects mitochondrial function (Figures 2A,B). VEGF co-treatment significantly improved maximal respiration rates, resulting in increased RCR when compared to ECs treated with PE serum (Figures 2A,B). Still, this improvement was not enough to reach the levels detected in cells treated with NOR serum. This scenario was similar in trophoblasts. PE serum caused a significant decrease in the maximal respiration rate and the addition of VEGF was not able to restore these values (Figure 2C). Then, the dramatic decrease in the spare respiratory capacity and RCR of cells treated with PE serum was partially restored by VEGF (Figures 2C,D). These observations suggested that sFlt-1 might be responsible for antagonizing the homeostatic activity of VEGF, having a direct impact on mitochondrial function and bioenergetics. However, it is expected that other factors in PE serum (and to a lesser extent in NOR and CTL women) are also contributing to the effects observed.

**Figure 3:**
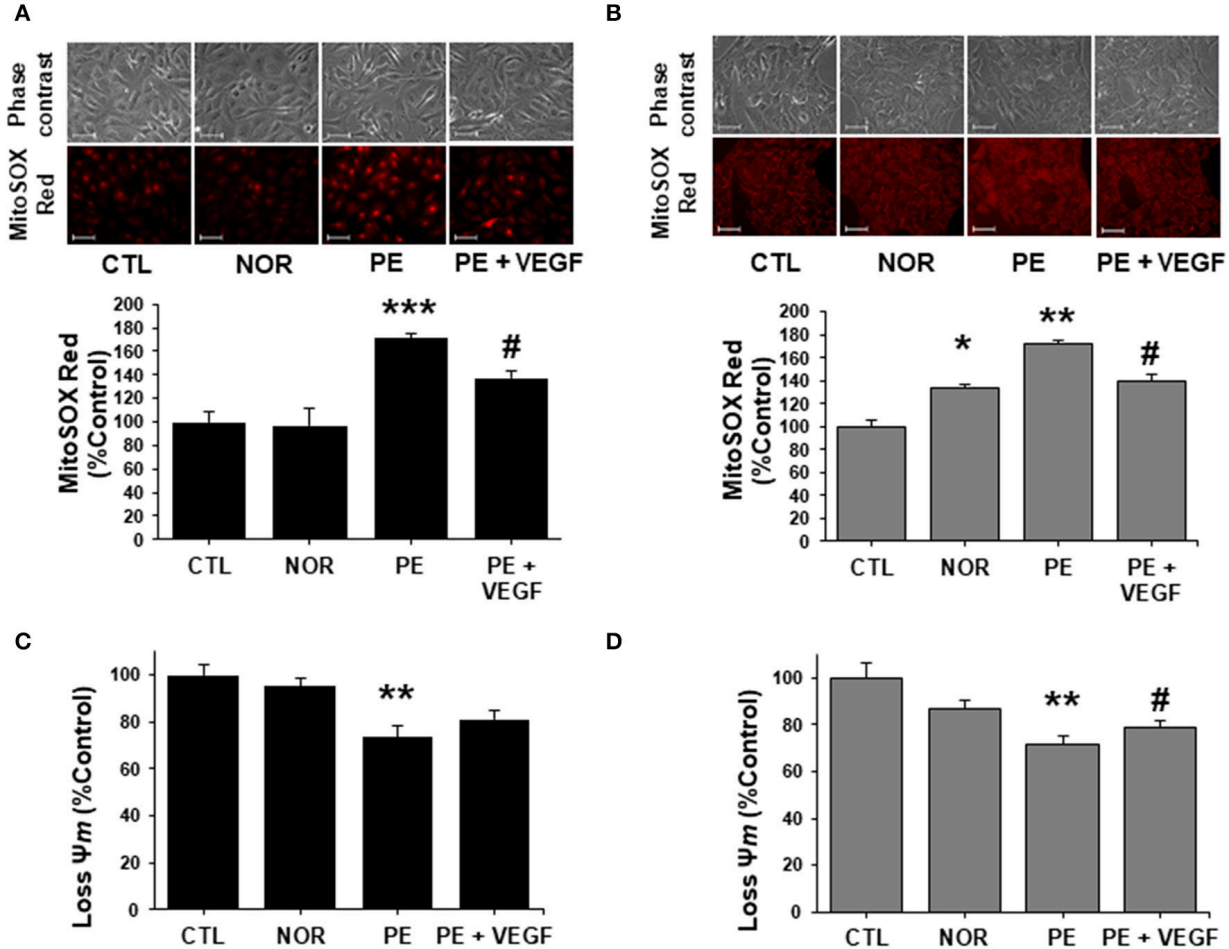
VEGF restores mitochondrial function and abrogates ROS in preeclampsia. ECs and HTR-8/SVneo cells were treated with 2% serum from NOR, CTL, and PE women with and without exogenous VEGF (20 ng/mL) for 24 h. **(A)** Mitochondrial ROS (mtROS) determinations by fluorescent microscopy using MitoSOX Red fluorescent probe in ECs. **(B)** As in **(A)** but with trophoblasts cells. **(C)** PE serum dissipated the mitochondrial membrane potential (Ψ*m*) measured by fluorescent microscopy using JC-1 fluorescent dye in ECs and **(D)** trophoblasts. VEGF (20 ng/mL) restored the mitochondrial membrane potential to CTL levels in trophoblasts. Scale: 100 μm. Data are presented as means ± SEM. (*n* = 3), ^*^P < 0.05, ^**^P < 0.01, ^***^P < 0.001, vs. CTL exposed cells. #*P* < 0.05, vs. PE serum exposed cells, ANOVA (*Bonferroni's post hoc test*).

Next, we examined the effects on ECs and trophoblasts of PE serum alone and with VEGF, in mitochondrial function, by assessing the production of mtROS with Mito-SOX fluorescence and ΔΨ*m* with JC-1 dye. In ECs, treatment with PE serum caused a drastic increase in mtROS in comparison with CTL and NOR serum. VEGF was only able to reduce mtROS generation by approximately 30% in comparison with ECs treated with PE serum (Figure 3A). In contrast, treatment in trophoblasts caused a significant increase as compared to CTL serum of 33% with NOR serum treatment and 71% increase in mtROS with PE serum (Figure 3B). VEGF decreased mtROS production about 30%, as in trophoblasts treated with NOR serum. PE serum also caused changes in the Ψ*m* in both ECs and HTR-8/SVneo cells. Treatment with PE serum caused a drop in Ψ*m* of about 27% in comparison to ECs treated with CTL serum (Figure 3C). Administration of VEGF was not able to restore the Ψ*m* as evidenced in the 8% increase in Ψ*m* in treated ECs. Similar results were obtained in treated trophoblasts (Figure 3D). Cells treated with PE serum experienced a drop in Ψ*m* of 28% in comparison with CTL serum. Treatment with PE serum and VEGF was only able to restore the drop in mitochondrial membrane potential with a 7% increase. In contrast, the effects of VEGF almost recover the changes in Ψ*m* to similar levels of cells treated with NOR serum.

### sFlt-1 induces a metabolic phenotype switch to glycolysis in ECs, but not in trophoblasts

**Figure 4:**
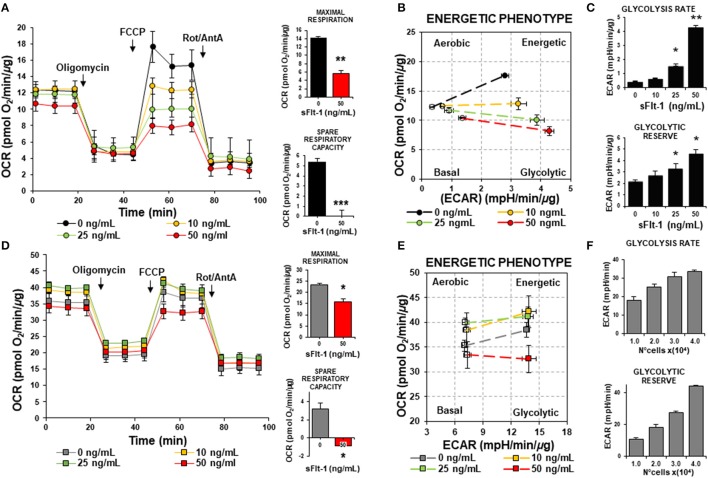
sFlt-1 induces a metabolic phenotype switch to glycolysis in ECs but not in trophoblasts. ECs and HTR-8/SVneo cells were exposed to 0, 10, 25, and 50 ng/mL of rh-sFlt-1 for 24 h. **(A)** Oxygen consumption rates (OCR), maximal respiration (OCR after FCCP administration) and spare respiratory capacity (Difference between basal and maximal OCR), **(B)** Energetic phenotype map, basal and maximal OCR and extracellular acidification rates (ECAR), **(C)** ECAR determinations of glycolysis rate and glycolytic reserve, evaluated in endothelial cells. **(D-F)**, are the same experiments as in **(A–C)**, respectively, but evaluated in HTR-8/SVneo cells. Data is presented as means ± SEM. (*n* = 5), ^*^P < 0.05, ^**^P < 0.01, ^***^P < 0.001 vs. untreated controls, In **(A,D)**: Student *T*-test. In **(C,F)**: ANOVA (*Bonferroni's post hoc test*).

Circulating levels of sFlt-1 are known to increase drastically prior the onset of clinical signs of PE (Maynard et al., 2003; Levine et al., 2004, 2006). To assess the effects of sFlt-1 on the mitochondrial bioenergetics of ECs and HTR-8/SVneo, cells were treated with sFlt-1 (0–50 ng/mL) for 24 h and subjected to OCR and ECAR measurements. As shown in Figure 4A, sFlt-1 decreased basal and ATP-dependent OCR in a dose-dependent manner in ECs. Also, significant changes in FCCP-stimulated OCR were shown in sFlt-1-treated cells, indicating that mitochondrial spare respiratory capacity was also affected (Figure 4A). These evidence demonstrate that sFlt-1-induced angiogenic imbalance, reduce the capacity of the endothelium to respond to changes in energy demand coupled to mitochondrial respiration (Supplementary Figure 3A). The analysis of OCR versus ECAR in sFlt-1-treated ECs showed an inhibition of the mitochondrial respiration, with a dose-dependent ability to switch to glycolysis (Figure 4B). In addition, the glycolytic response increased more than 5-fold in 50 ng/mL sFlt-1-exposed cells, suggesting a metabolic phenotype switch to glycolysis in ECs, after treatment with sFlt-1 (Figure 4C and Supplementary Figure 3B). Regarding HTR-8/SVneo cells, cells demonstrated a higher basal OCR with an overall reduced FCCP-dependent OCR, in comparison to ECs, displaying high glycolytic rates and glycolytic reserves even at basal conditions (Figures 4E,F). Response to sFlt-1 treatment showed a reduction in mitochondrial respiration without increases in the glycolytic flux exerted by 50 ng/mL of sFlt-1 (Figures 4D–F).

Results obtained clearly state that in both, ECs and HTR-8/SVneo, sFlt-1 is acting as a disruptor directly into mitochondria. Nonetheless, based on the differential cell energy phenotypes of ECs and HTR-8/SVneo, the effects are interestingly dissimilar in both cell lines.

### sFlt-1 acts as a mitochondrial bioenergetics disruptor in ECs and HTR-8/SVneo

To further confirm the effects of sFlt-1 as a mitochondrial bioenergetics disruptor, mitochondrial metabolism (OXPHOS) was forced by growing cells in galactose media (Robinson et al., 1992; Marroquin et al., 2007). This approach forces cells to exclusively rely on mitochondrial metabolism of glutamine for their ATP energy requirements (Reitzer et al., 1979). Reliance on OXPHOS for ATP production, sensitize cells to mitochondrial disruptors(Marroquin et al., 2007; Dott et al., 2014).

**Figure 5:**
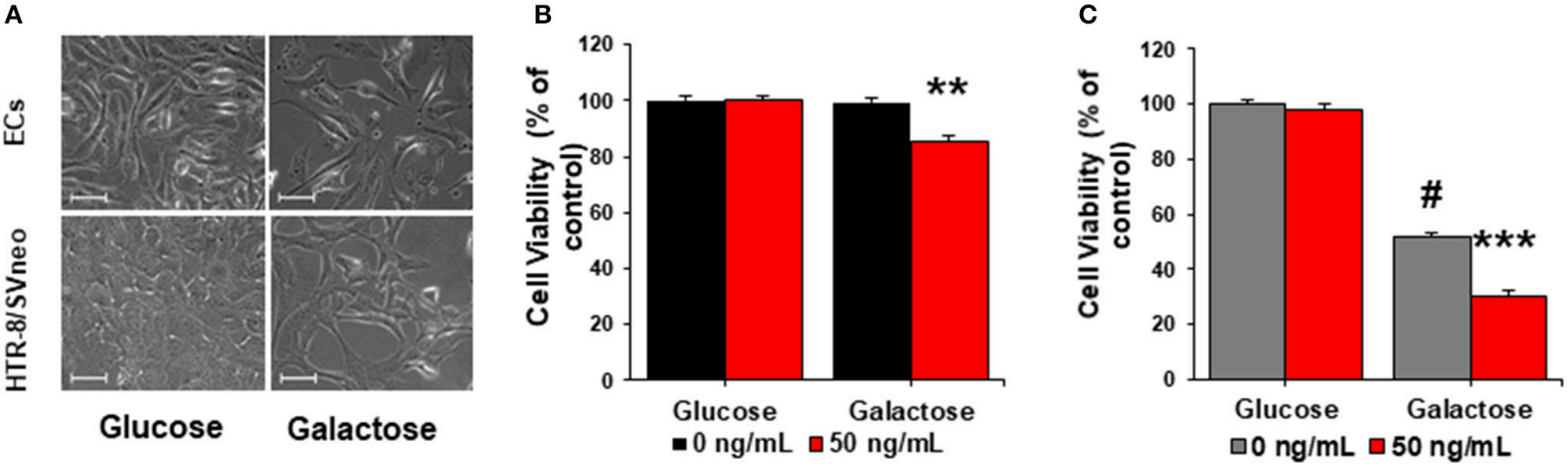
sFlt-1 acts as a mitochondrial bioenergetics disruptor in preeclampsia. **(A)** Morphological changes in endothelial cells (ECs) and trophoblasts cultured in glucose and galactose media (40X). **(B)** Cell viability of ECs and **(C)** trophoblasts cultured in glucose and galactose media and exposed to exogenous 50 ng/mL of sFlt-1 during 24 h. Scale: 100 μm. Data are presented as means ± SEM. (*n* = 3), ^**^P < 0.01, ^***^P < 0.001, vs. galactose exposed cells. ^#^*P* < 0.05, vs. glucose-exposed cells. Student *T*-test.

ECs and HTR-8/SVneo exposed to galactose presented slower growth rates in comparison to cells cultured in glucose media (Figure 5A). ECs, cultured in galactose, did not evidence changes in their viability or proliferations rates in comparison to cells exposed to glucose (Figure 5B and Supplementary Figure 4A). Nonetheless, when ECs were exposed to sFlt-1 in galactose media, they had significant reduction in the proliferation rate and viability of about 20% in comparison to non-treated cells (Figure 5B and Supplementary Figure 4A).

Divergently, the culture of HTR-8/SVneo cells in galactose, evidenced their dependence on glycolysis as a main source of energy, as described previously (Figures 4E,F). By forcing OXPHOS in HTR-8/SVneo cells, we confirmed diminished proliferation and viability rates of about 50% in comparison to cells grown in glucose (Figure 5C and Supplementary Figure 4B). These observations are consistent with HTR-8/SVneo evolutionary metabolism of a first-trimester trophoblast, previously defined to be contingent mainly on non-oxidative pathways to support its energetic demands (Illsley et al., 2010). Thus, treatment of HTR-8/SVneo cells with sFlt-1 in galactose media evidenced a reduced viability of about 40% in comparison to controls in galactose (Figure 5C and Supplementary Figure 4B). Together, these results evidenced that sFlt-1, as a mitochondrial disruptor, affects more dramatically cells that rely on mitochondria machinery for energy proposes, rather than those with a basal glycolytic dependence for their metabolism.

**Figure 6:**
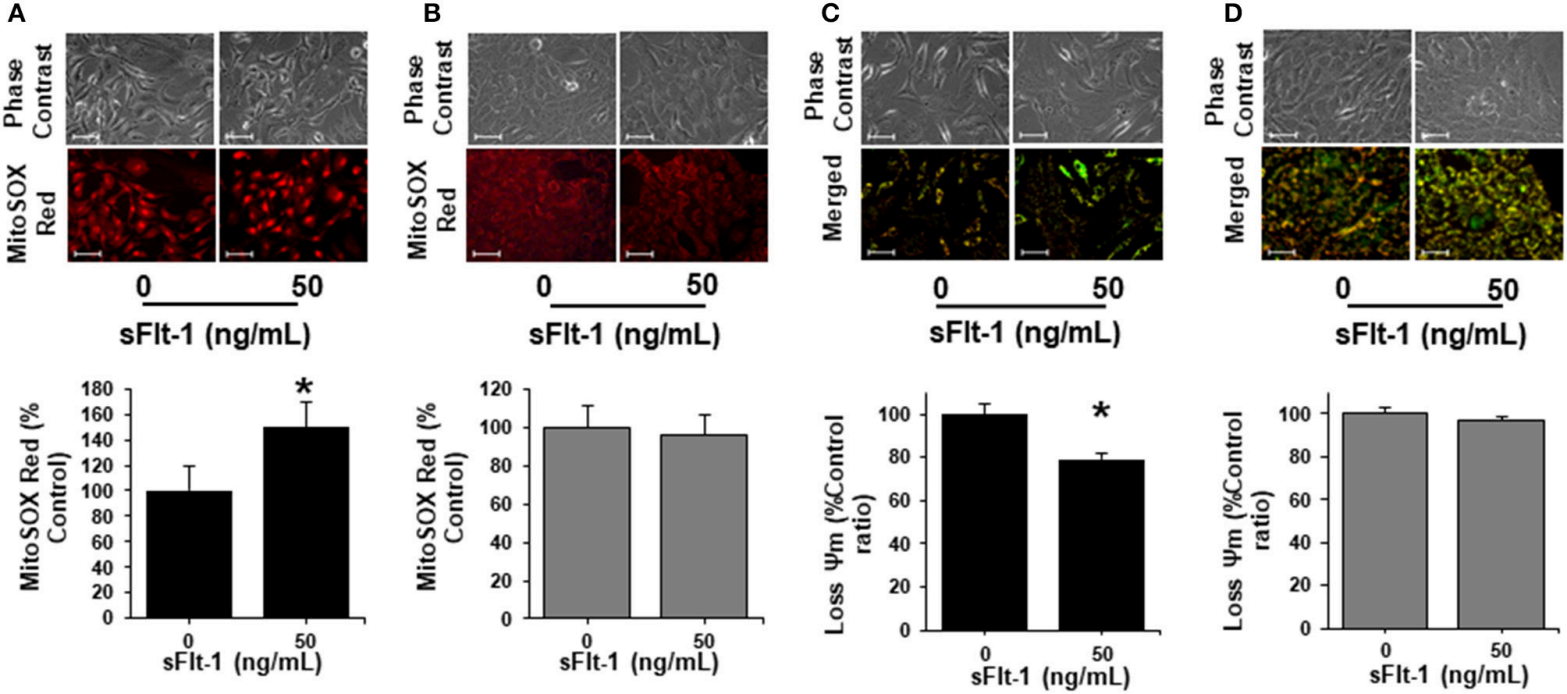
sFlt-1 induces mitochondrial dysfunction *in vitro*. **(A)** Mitochondrial ROS (mtROS) determinations by fluorescent microscopy using MitoSOX Red fluorescent probe demonstrate that sFlt-1 significantly induced mtROS formation in endothelial cells (ECs), while these effects were not observed in **(B)** trophoblasts. **(C)** sFlt-1 dissipated the mitochondrial membrane potential (Ψ*m*) measured by fluorescent microscopy using JC-1 fluorescent probe in ECs, but not in **(D)** trophoblasts. Cells were exposed to sFlt-1 (50 ng/mL) for 24 h. Data is presented as means ± SEM. (*n* = 3), ^*^P < 0.05 vs. untreated controls. Student *T*-test.

To further corroborate the impact of sFlt-1 over the mitochondrial function and ROS formation, we evaluated the production of mtROS and ΔΨ*m* in ECs and trophoblasts, as measured by fluorescence microscopy. As shown in Figure 6A, using fluorescent probe MitoSOX Red, we evidenced that sFlt-1 (50 ng/mL) significantly increased mtROS formation in ECs 1.5-fold, but these effects were not observed in trophoblasts (Figure 6B). Then, the evaluation of the ΔΨ*m* using JC-1 fluorescent dye, established that doses of 50 ng/mL of exogenous sFlt-1, reduced Ψ*m* in ECs by 20% (Figure 6C). Again, no significant changes were found in trophoblast cells (Figure 6D). These results confirm our previous observations regarding the role of sFlt-1 to induce alterations in mitochondrial bioenergetics in ECs and its impact on the overall mitochondrial function, as previously demonstrated *in vivo* (Jiang et al., 2015).

## Discussion

PE remains as the leading cause of maternal and neonatal deaths worldwide (Duley, 2009). Within the last decade, several reports opened a novel window for understanding the pathogenesis of the disease, by describing the role of circulating anti-angiogenic factors like sFlt-1, in the development and early detection of the disease (Maynard et al., 2003; Levine et al., 2004; Young et al., 2010; Perni et al., 2012; Verlohren et al., 2012). sFlt-1 has been found to provoke endothelial dysfunction (Powe et al., 2011; Sánchez-Aranguren et al., 2014), hypertension (LaMarca et al., 2008) and proteinuria (Maynard and Karumanchi, 2011), demonstrating its culprit role in the onset of PE (Roberts and Rajakumar, 2009). The present study demonstrates the striking effects of sFlt-1 and serum from PE women in the overall cell energy metabolism, mitochondrial bioenergetics and mitochondrial dysfunction, linked to oxidative stress in ECs and first trimester EVT (HTR-8/SVneo cells). Early metabolic perturbations are known to be the hallmark of a range of human pathologies (DeBerardinis and Thompson, 2012). Then, the study of cellular energy metabolism arrives a novel strategy for understanding the etiology of diseases and potentially to develop novel treatments targeting these alterations in energy metabolism (Gohil et al., 2010).

### sFlt-1, increased in PE serum, induce metabolic perturbations in preeclampsia

By the aid of the Seahorse-Agilent Extracellular Flux Analyzer, we evaluated the metabolic perturbations preceding the onset of PE. In order to establish a model that resemble PE conditions, ECs and HTR-8/Svneo cells were treated with serum from pregnant women, normotensive and preeclamptic, based on current diagnostic criteria (American College Obstetricians Gynecologist Task Force on Hypertension in Pregnancy, 2013). ELISA analyses showed increased sFlt-1 levels in serum from PE women by several orders of magnitude, in comparison with NOR and CTL serum levels, as reported previously (Maynard et al., 2003; Levine et al., 2004, 2006).

In a normal pregnancy, circulating sFlt-1 levels increase with gestation age (Maynard et al., 2005). However, in PE, due to unknown mechanisms, sFlt-1 is dramatically upregulated (Fan et al., 2014), resulting in increased circulating levels of sFlt-1 prior the onset on the clinical signs of PE (Maynard et al., 2003; Levine et al., 2004, 2006). Here, we have demonstrated and described the alterations in the mitochondrial bioenergetics profiles induced by sFlt-1 increased levels, in both cell lines tested. Our results are in accordance with recent work showing that incubation of HUVEC with 3% plasma from PE women, significantly reduced the overall OCR, measured by a fluorescence-based approach, when compared to cells treated with serum from uncomplicated and non-pregnant women (McCarthy and Kenny, 2016). In addition, our results demonstrate the culprit role of sFlt-1 in the onset of metabolic alterations in PE. sFlt-1 might be responsible for antagonizing the homeostatic activity of VEGF, having an impact at the mitochondrial level. However, it is likely that other molecules, existing in the maternal serum of PE women, could be potentiating the effects observed.

We also showed that mtROS are increased in trophoblasts exposed to NOR serum in comparison to cells exposed to CTL serum. This fact is consistent with the increased oxidative stress status at the intrauterine level, observed by others in normal pregnancies (Chaiworapongsa et al., 2014). PE serum, in ECs and trophoblasts, led to a significant increase in mtROS production in comparison to cells exposed to NOR serum. The augmented mitochondrial oxidative stress, evidenced an alteration in mitochondrial function induced by elevated sFlt-1 levels, present in PE serum. It is likely that increased mtROS production would be directly related to the effects of sFlt-1 on mitochondrial bioenergetics. As it has been reported previously *in vivo*, sFlt-1 induces ROS formation in placental vessels and trophoblasts (Jiang et al., 2015). Potential mechanisms of increased mtROS formation have been associated to a reverse electron transport through complex I, in the mitochondrial electron transport chain, in response to an elevated NADH/NAD^+^ ratio (Murphy, 2009). Our observations suggest that dysregulated sFlt-1 levels during pregnancy, induce metabolic perturbations and oxidative stress that might contribute to vascular endothelial dysfunction in PE.

### sFlt-1 induce a metabolic phenotype switch to glycolysis in endothelial cells

To better understand the metabolic bases that would accompany the onset of PE, we studied the role of increasing concentrations of sFlt-1, as a potential early metabolic disturber. We showed a dose-dependent loss of mitochondrial function in ECs treated with sFlt-1. Treatment with increasing concentrations of sFlt-1, evidenced a metabolic phenotype switch from OXPHOS to an enhanced glycolytic cellular response, not been establish before. Our data suggest that in PE, increasing sFlt-1 levels could result in loss of mitochondrial function early in gestation, leading to impaired bioenergetics profiles. Cell energy metabolism varies in tissue of different origins (Benard et al., 2006). Therefore, cell metabolism can be adapted according to the microenvironment surrounding cells. In evidence, other authors have reported that various growth conditions may alter metabolism, contributing to a greater cell dependence on glycolysis (Jose et al., 2011). Our observations of an enhanced glycolytic metabolism, along with an impaired oxygen consumption, suggested a metabolic reprogramming process, as described in tumor biology studies (Jose et al., 2011). These observations illustrate how ECs can alternate OXPHOS to glycolytic pathways under sFlt-1-induced stressful conditions. Nonetheless, the potential effects derived from the prolongation of a aerobic glycolytic metabolism (Warburg effect) in ECs, remains to be determined. Enhanced glycolysis linked to a reduced OXPHOS could markedly affect ECs capabilities to switch from a quiescent metabolic state to an energetic phenotype during angiogenesis.

Regarding HTR-8/SVneo cells, we found, as reported before (Illsley et al., 2010), that they have a functional mitochondrial machinery. In contrast to ECs, sFlt-1 treatment in trophoblasts, causes a non-significant mild drop in OCR. Since the cell basal energetic profile is glycolytic, the overall energetic phenotype after sFlt-1 treatment was not impaired. Studies performed on term isolated trophoblasts have shown that both, syncytium and cytotrophoblast cells, exhibit high reserve respiratory capacity (Maloyan et al., 2012) when compared with our results. This suggests that trophoblast cells, in early stages of gestation, are metabolically programmed to overlap and compensate for the effects of metabolic disruptors. Based on these facts, we presumed that in later stages of pregnancy, when placental function reaches its inevitable end, the metabolic profile of trophoblasts changes as their biological functions terminate. These suggest that cells isolated from full-term placentas may not be the most appropriate approach to study the effects of early metabolic perturbations.

### sFlt-1 acts a mitochondrial disruptor

Our results demonstrate a dose-dependent alteration in the mitochondrial bioenergetics, suggesting that sFlt-1 is acting as mitochondrial disruptor. To demonstrate the ability of sFlt-1 to act as a mitochondrial disruptor that drives energy metabolism from OXPHOS to glycolysis, we forced ATP dependence on OXPHOS, by culturing cells in galactose media. Since oxidation of galactose to pyruvate through glycolysis yields no net production of ATP, cells are more sensitive to mitochondrial toxicants, than when grown in glucose (Marroquin et al., 2007). First, culture of ECs in galactose did not involve changes in proliferation rates or viabilities, consistent with their metabolic flexibility to switch from ATP predominantly generated by OXPHOS, to glycolysis as their main energy source (Vallerie and Bornfeldt, 2015). sFlt-1 impaired ECs viability and proliferation rates under OXPHOS dependence, demonstrating that cells that rely mainly in mitochondrial metabolism are highly sensitive to sFlt-1. Results were markedly drastic in trophoblasts. Galactose media enhanced the effects of sFlt-1, decreasing cell viability and proliferation rates of about 40%. In both cell lines tested, culture in galactose media overblown sFlt-1 effects, demonstrating its role as a mitochondrial disrupter, effects that are not basally appreciated in glucose media. Previously, culture in galactose has been employed to identify mitochondrial toxicants (Dott et al., 2014) and molecules that target cellular metabolic shifts as potential therapeutics for pathologies associated with ischemia/reperfusion damage (Gohil et al., 2010).

**Figure 7:**
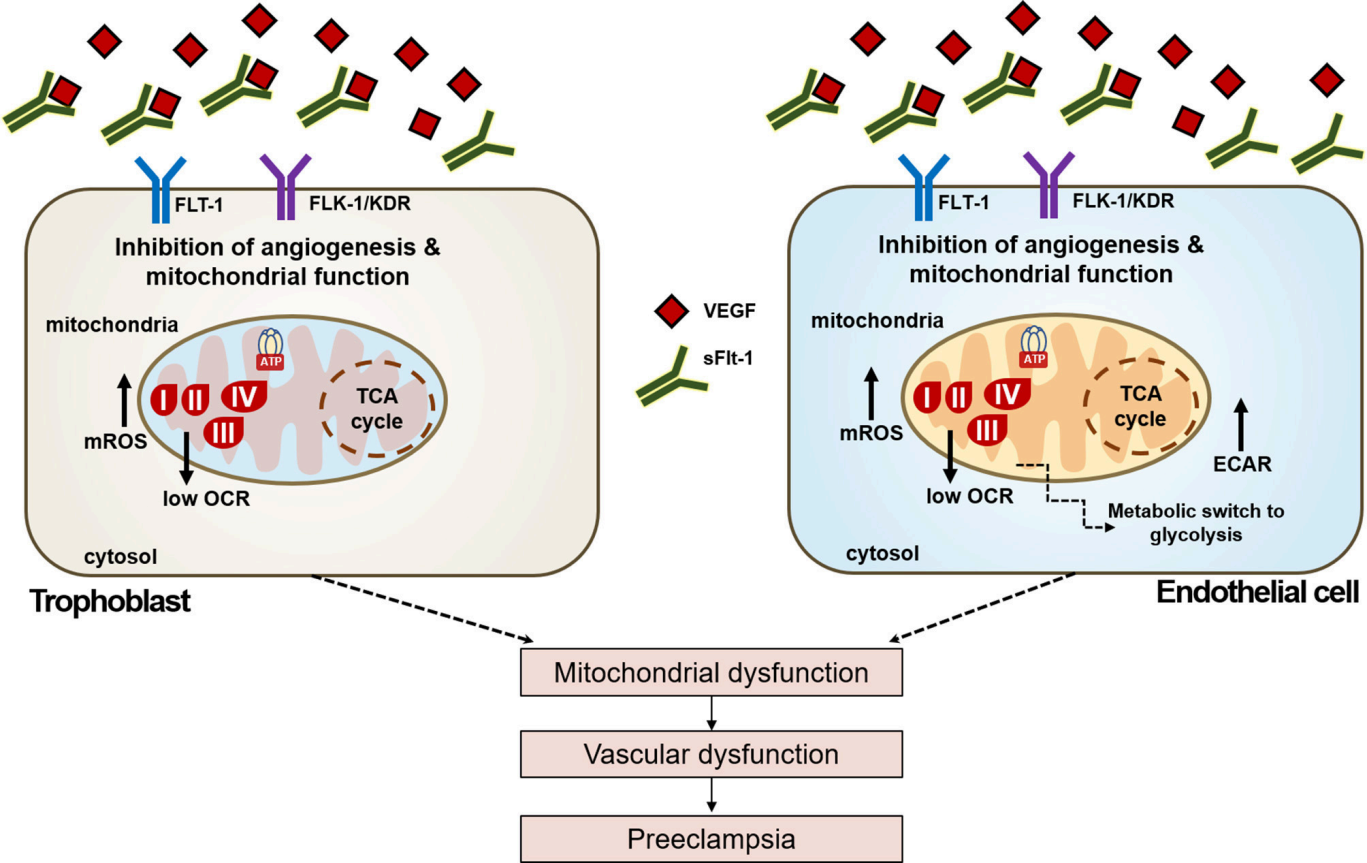
Schematic view of the effects of sFlt-1 dysregulation over cellular metabolism and bioenergetics in PE. Dysregulated VEGF signaling due to up-regulation of sFlt-1 levels in preeclampsia leads to reduced activation of VEGF receptors Flt-1 and Flk-1/KDR, respectively. Effects of dysregulated VEGF bioavailability affect mitochondrial oxygen consumption (OCR), inducing a metabolic phenotype switch enhancing glycolytic response (ECAR) in endothelial cells, but not in trophoblasts. sFlt-1 due to loss of mitochondrial bioenergetics increase oxidative stress in mitochondria (mtROS). Together, these events lead to mitochondrial dysfunction, that would result in vascular dysfunction and the onset of preeclampsia.

The increased mtROS production and decreased mitochondrial respiration, coupled with a higher glycolytic capacity of ECs exposed to sFlt-1, evidenced oxidative stress and a metabolic phenotype switch to compensate the detrimental effects of sFlt-1 and mediators present in serum from PE women, over the endothelium. Our results demonstrated that exogenous sFlt-1 induce mtROS formation and a drop in the mitochondrial membrane potential in ECs, but not in trophoblasts. This suggested that sFlt-1 plays a key role in metabolic modulation and reprogramming in endothelium during pregnancy. Whereas, when sFlt-1 levels increase drastically, their role is balanced toward an anti-angiogenic state that leads to metabolic impairment, vascular dysfunction, and PE. sFlt-1 seems to be an important linker between mitochondrial dysfunction, oxidative stress and endothelial dysfunction (Figure 7).

Here we have demonstrated that in trophoblast and endothelial cells, disruption of the finely-tuned VEGF signaling by sFlt-1 affects mitochondrial function and metabolism in preeclampsia. As Figure 7 reviews, sFlt-1 strongly impairs mitochondrial metabolism and bioenergetics, increasing mROS and inducing a metabolic switch to glycolysis in ECs. These findings are very important because they confirm the differential responses of sFlt-1 in both ECs and trophoblasts that are directly related in the development of the disease, from the maternal circulation and placenta, respectively. In addition, results obtained in ECs have strong implications in the maternal hypertension events that are the hallmark of PE. Various reports have revealed the direct implication of VEGF signaling in the regulation of mitochondrial function and angiogenesis (Wang et al., 2011; Guo et al., 2017; Kim et al., 2017). However, the exact mechanisms on how VEGF and downstream events regulate mitochondrial function are still unknown. Nevertheless, the role of the PI3k/Akt/mTOR pathways and eNOS and NO production in relation to high levels of sFlt-1, VEGF signaling, and mitochondrial function is currently under study. Establishing the clear role of sFlt-1/VEGF signaling in PE is key for developing novel strategies for preventing or treating this multifactorial disease.

## Author contributions

LS-A and ML: conceived and designed the experiments with the help from CE-G, LG-O, and SS-B; CR-M and AN provided serum samples and clinical expertise; LS-A: drafted the manuscript. Analysis was conducted by LS-A and ML; LS-A, AA, JV-V, and ML: read/revised and approved the final manuscript.

### Conflict of interest statement

The authors declare that the research was conducted in the absence of any commercial or financial relationships that could be construed as a potential conflict of interest.

## Supplementary material

Supplementary Figure 1 Parameters of mitochondrial bioenergetics and cellular metabolism in intact cells. **(A)** Oxygen consumption rates (OCR) determinations and parameters of mitochondrial function. **(B)** Energetic phenotype map shows the metabolic state of cells and **(C)** extracellular acidification rates (ECAR) determination and parameters of the glycolytic function.

Supplementary Figure 2 sFlt-1 levels measured by ELISA. Serum levels were measure in non-pregnant controls (CTL), normotensive (NOR) and preeclamptic (PE) women by ELISA. Data is presented as means ± SEM. (*n* = 6), ^*^*P* < 0.05 vs. CTL, ^#^*P* < 0.05 vs. NOR. ANOVA (Bonferroni's *post hoc* test).

Supplementary Figure 3 sFlt-1 induced mitochondrial bioenergetics dysfunction *in vitro*. **(A)** Ratios of basal and maximal (Max) respiratory control (RCR) (State 3/state 4) and **(B)** Extracellular acidification rates (ECAR) yield by time, demonstrate a metabolic phenotype switch from mitochondrial phosphorylation to glycolysis, in endothelial cells exposed to 0, 10, 25, and 50 ng/mL of exogenous sFlt-1 for 24 h. Data is presented as means ± SEM (*n* = 5), ^*^*P* < 0.05, ^**^*P* < 0.01, ^***^*P* < 0.001 vs. maximal RCR measured in untreated controls. ANOVA (Bonferroni's *post-hoc* test).

Supplementary Figure 4 sFlt-1 acts as a mitochondrial bioenergetics disruptor. **(A)** Cell proliferation counts measured in endothelial cells and **(B)** trophoblasts cultured in glucose and galactose media and also exposed to 50 ng/mL of exogenous sFlt-1 for 24, 48, and 72 h. Data is presented as means ± SEM. (*n* = 3), ^*^*P* < 0.05, vs. galactose exposed cells. ^#^*P* < 0.05, vs. glucose exposed cells. ANOVA (Bonferroni's *post-hoc* test).

Supplementary Table 1 Clinical characteristics of the recruited women. Data is presented as means ± SEM. Primiparous variable values are presented in %. ^*^*P* = < 0.05, vs. non pregnant controls (CTL), ^#^*P* < 0.01 vs. NOR group. N.A. Data not available.
