# Supplementary figures and images for: Soluble Fms-Like Tyrosine Kinase-1 Alters Cellular Metabolism and Mitochondrial Bioenergetics in Preeclampsia
Source: Front Physiol. 2018 Mar 6;9:83. doi: 10.3389/fphys.2018.00083 (PMC5845757; doi:10.3389/fphys.2018.00083)

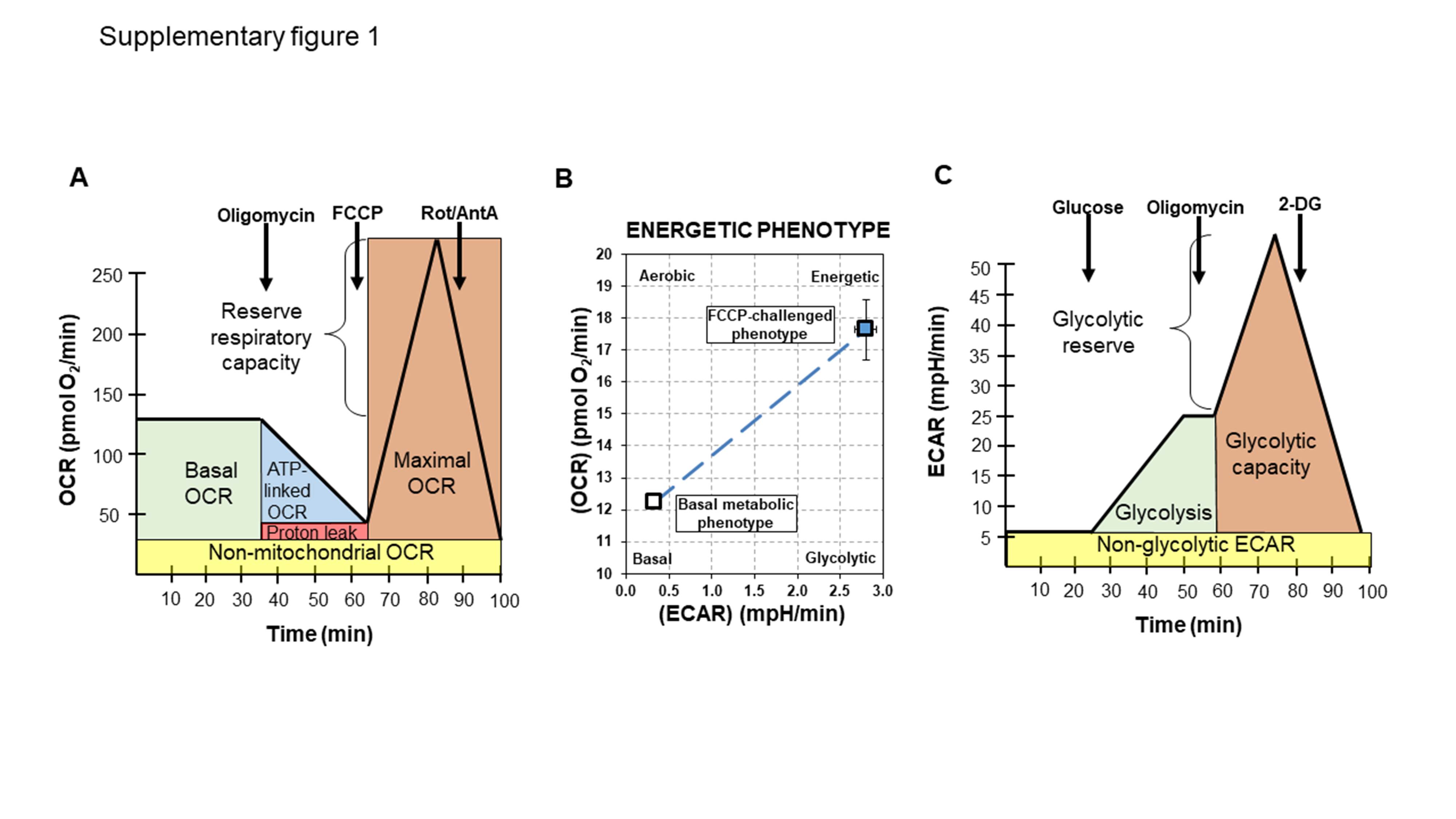

Supplement: Supplementary Figure 1 — Parameters of mitochondrial bioenergetics and cellular metabolism in intact cells. (A) Oxygen consumption rates (OCR) determinations and parameters of mitochondrial function. (B) Energetic phenotype map shows the metabolic state of cells and (C) extracellular acidification rates (ECAR) determination and parameters of the glycolytic function. [file Image1.TIF]

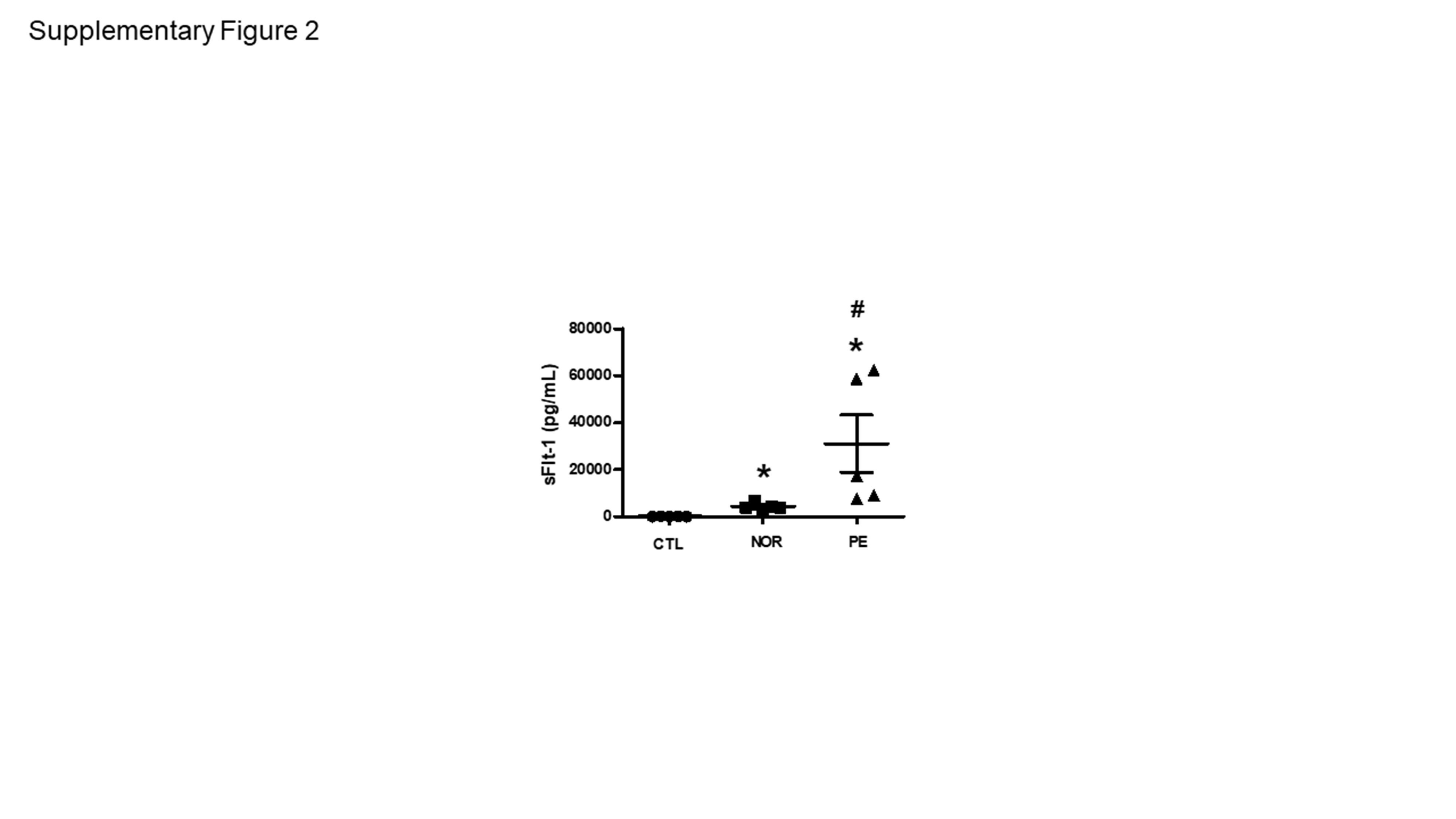

Supplement: Supplementary Figure 2 — sFlt-1 levels measured by ELISA. Serum levels were measure in non-pregnant controls (CTL), normotensive (NOR) and preeclamptic (PE) women by ELISA. Data is presented as means ± SEM. (n = 6), *P < 0.05 vs. CTL, #P < 0.05 vs. NOR. ANOVA (Bonferroni's post hoc test). [file Image2.TIF]

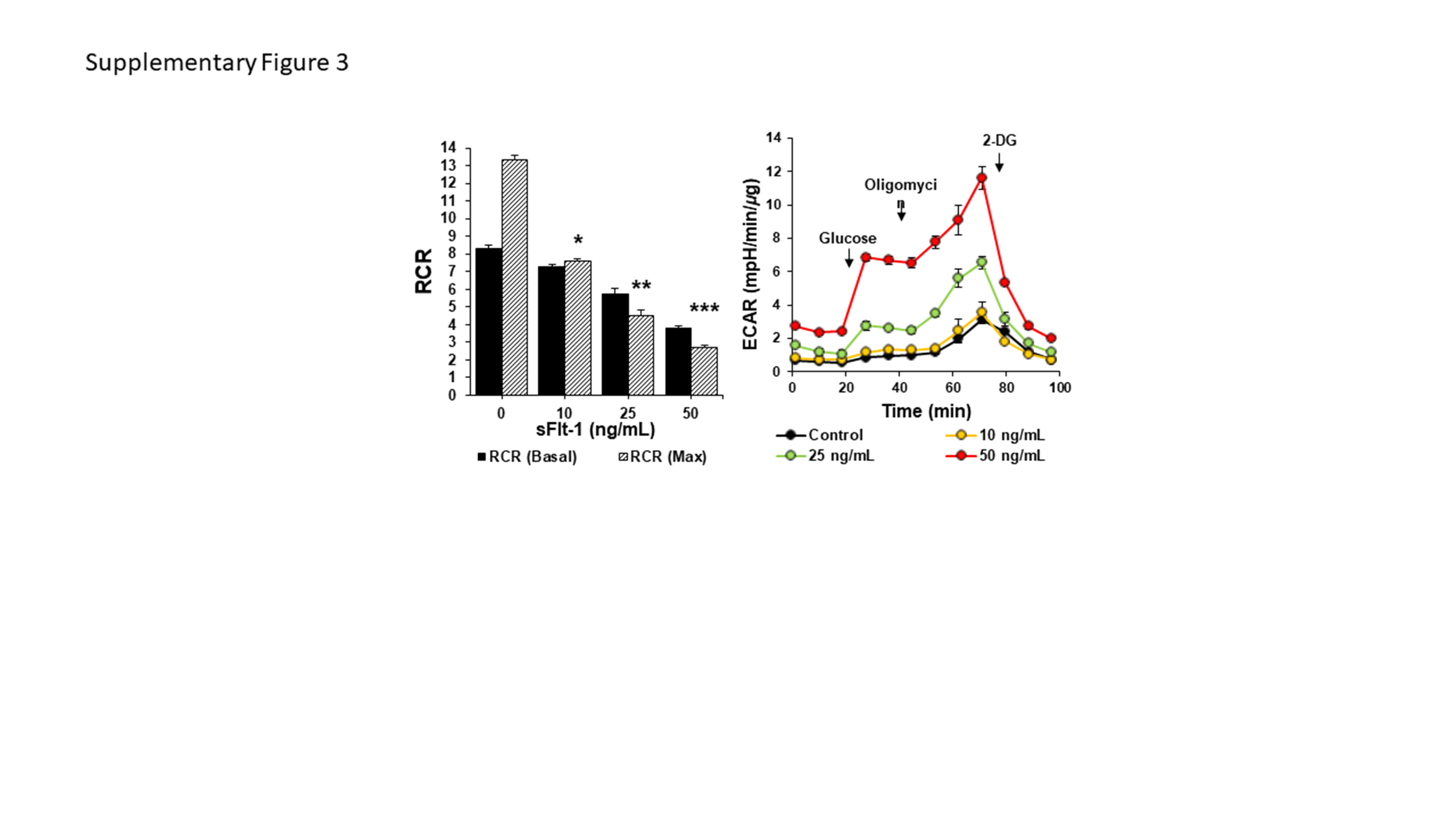

Supplement: Supplementary Figure 3 — sFlt-1 induced mitochondrial bioenergetics dysfunction in vitro. (A) Ratios of basal and maximal (Max) respiratory control (RCR) (State 3/state 4) and (B) Extracellular acidification rates (ECAR) yield by time, demonstrate a metabolic phenotype switch from mitochondrial phosphorylation to glycolysis, in endothelial cells exposed to 0, 10, 25, and 50 ng/mL of exogenous sFlt-1 for 24 h. Data is presented as means ± SEM (n = 5), *P < 0.05, **P < 0.01, ***P < 0.001 vs. maximal RCR measured in untreated controls. ANOVA (Bonferroni's post-hoc test). [file Image3.TIF]

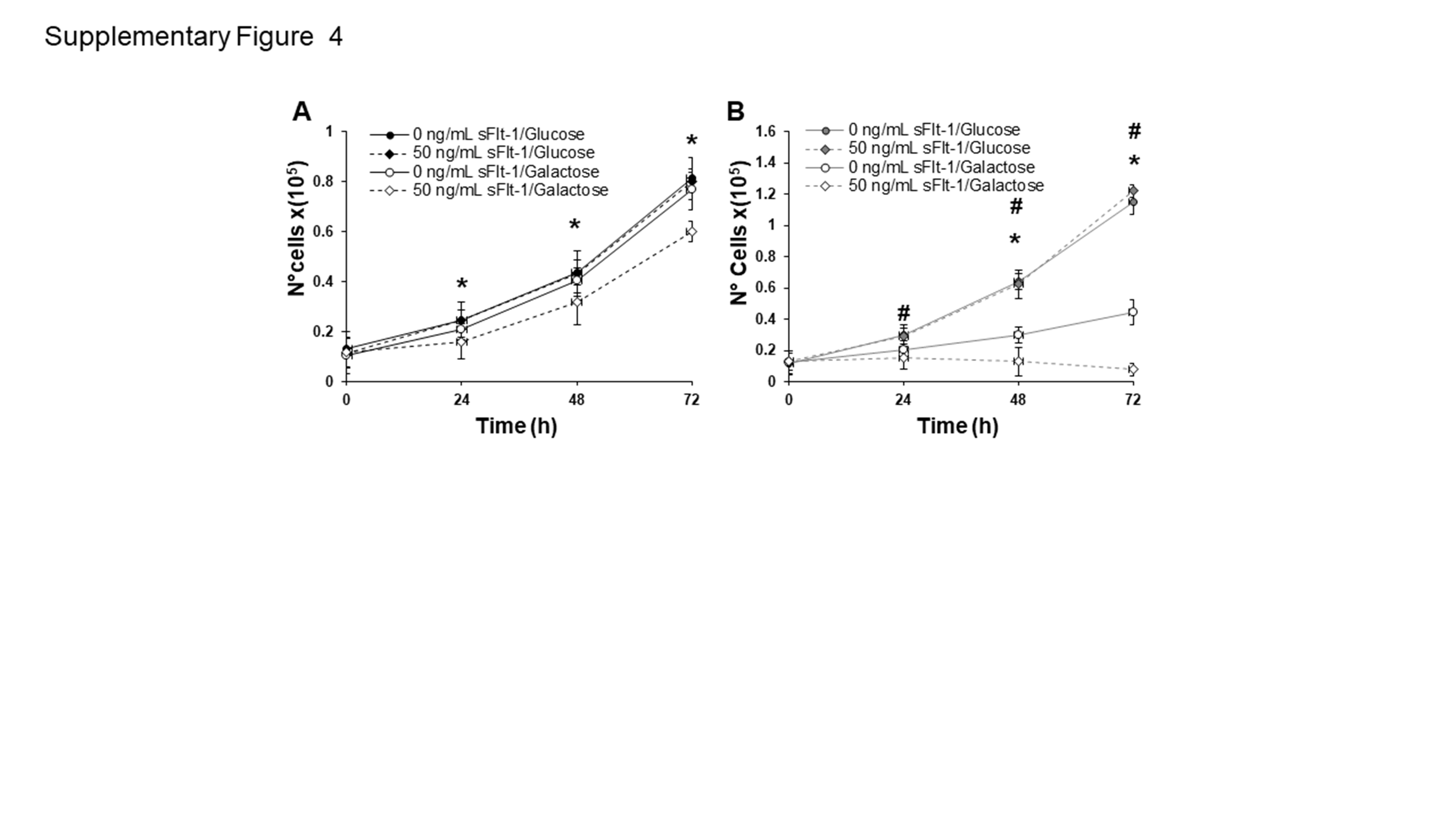

Supplement: Supplementary Figure 4 — sFlt-1 acts as a mitochondrial bioenergetics disruptor. (A) Cell proliferation counts measured in endothelial cells and (B) trophoblasts cultured in glucose and galactose media and also exposed to 50 ng/mL of exogenous sFlt-1 for 24, 48, and 72 h. Data is presented as means ± SEM. (n = 3), *P < 0.05, vs. galactose exposed cells. #P < 0.05, vs. glucose exposed cells. ANOVA (Bonferroni's post-hoc test). [file Image4.TIF]
