# Supplementary material for: Soluble Fms-Like Tyrosine Kinase-1 Alters Cellular Metabolism and Mitochondrial Bioenergetics in Preeclampsia
Source: Front Physiol. 2018 Mar 6;9:83. doi: 10.3389/fphys.2018.00083 (PMC5845757; doi:10.3389/fphys.2018.00083)
Supplement: Supplementary Table 1 — Clinical characteristics of the recruited women. Data is presented as means ± SEM. Primiparous variable values are presented in %. *P = < 0.05, vs. non pregnant controls (CTL), #P < 0.01 vs. NOR group. N.A. Data not available. [file Table1.DOCX]

| Variable | Subject Groups | | | ***P*** |
| --- | --- | --- | --- | --- |
|  | Non-pregnant (CTL) n=10 | Normotensive (NOR) n=23 | Preeclamptic (PE) n=20 |  |
| Maternal age (years) | 24±1.5 | 29.64±1.4 | 26.84±2.2 | 0.33* |
| Gestational age (weeks) | N.A. | 38.3±0.3 | 33.3±1.3# | <0.001# |
| Body mass index (BMI) (kg/m^2^) | 22.3±1.1 | 27.9±0.9 | 32.3±1.4*# | 0.03* |
| Primiparous (%) | N.A. | 12% | 65%# | <0.001# |
| Sistolic blood pressure (mmHg) | 112±1.3 | 115±1.9 | 150±6.8# | <0.001# |
| Diastolic blood pressure (mmHg) | 68±1.5 | 74±1.9 | 98±4.0# | <0.01# |
| Proteinuria (mg protein/24 h urine) | <300 | <300 | 458±32# | <0.001# |
