# Supplementary material for: Soluble Fms-Like Tyrosine Kinase-1 Alters Cellular Metabolism and Mitochondrial Bioenergetics in Preeclampsia
Source: Front Physiol. 2018 Mar 6;9:83. doi: 10.3389/fphys.2018.00083 (PMC5845757; doi:10.3389/fphys.2018.00083)
Supplement: Supplementary file 6 [file DataSheet1.docx]

Supplementary Material

Soluble fms-like Tyrosine Kinase-1 Alters Cellular Metabolism and Mitochondrial Bioenergetics in Preeclampsia

Lissette Carolina Sánchez-Aranguren^1,2^, Cindy Tatiana Espinosa-González^2^, Laura María González-Ortiz^1,2^, Sandra Milena Sanabria-Barrera^1,2^, Carlos Riaño-Medina^2,3^, Andrés Felipe Nuñez^4^, Asif Ahmed^5^, Jeannette Vásquez-Vivar^6^ and Marcos López^1,2, *^

^1^Traslational Biomedical Research Group, Fundación Cardiovascular de Colombia, Floridablanca, Santander, Colombia.

^2^Graduate Program in Biomedical Sciences, Faculty of Health, Universidad del Valle, Cali, Colombia

^3^Maternal-Fetal Medicine Unit, Fundación Cardiovascular de Colombia, Floridablanca, Santander, Colombia.

^4^Maternal-Fetal Medicine Unit, Clínica Materno Infantil San Luis, Bucaramanga, Santander, Colombia.

^5^Aston Medical Research Institute, Aston Medical School, Aston University, Birmingham, UK

^6^Redox Biology Program and Free Radical Research Center, Department of Biophysics, Medical College of Wisconsin, Milwaukee, Wisconsin, United States.

***Correspondence:**Corresponding Author: Marcos Lopez, Ph.D. Translational Biomedical Research Group. Fundación Cardiovascular de Colombia. Carrera 5a No. 6-33, Floridablanca, Santander, Colombia, 681003, Telephone: +57 316 481 9276; e-mail: [marco4357@gmail.com](mailto:marco4357@gmail.com)

# Supplementary Data

## Blood Sample Collection

Antecubital blood samples from recruited women were collected minutes before delivery (pregnant normotensive and preeclamptic patients), according to institutional guidelines. Samples were maintained at room temperature before centrifugation at 1500*g* for 10 min to allow clot formation. Then, aliquoted under sterile conditions and stored at −80°C until use.

## Cell proliferation assays

Trypan blue exclusion assays were performed as cell proliferation assays. A uniform suspension of cells was seeded on 24-well plates. Cells were treated with 50 ng/mL of sFlt-1 recombinant protein (R&D systems). Replicates samples were count from 0-72 h. ECs and HTR-8/SVneo were cultured using glucose and galactose supplemented media. Mean values of three independent counts were used for the analysis.

## ELISA

Serum from recruited women was used for ELISA determinations of sFlt-1 levels. Human VEGF-R1/Flt-1 Quantikine ELISA Kits were employed as manufacturer’s instructions (R & D Systems).

## Agilent-Seahorse XFe24 Technology

Extracellular flux analysis use label-free systems that allow the detection in real time, of changes in cell bioenergetics of adherent cells in culture and tissue explants. The XF Analyzer is a powerful tool that simultaneously measure oxygen consumption rates (OCR) and extracellular acidification rates (ECAR), which can be correlated to oxidative phosphorylation and glycolysis, respectively. The XF Analyzer measures OCR and ECAR values in the surrounding media of a monolayer of cells, seeded in Seahorse V7 24 well microplates. Also, the Analyzer has injection ports attached to each well of the microplate, that allow the injection of inhibitors/activators of the mitochondrial respiratory chain and glycolysis, that let to determine alterations in cellular respiration and glycolytic response.

### Mitochondrial oxygen consumption

To evaluate the mitochondrial function, ECs and HTR-8/SVneo cells were seeded in V7 Seahorse micro-well plates at 3.5-4.0 × 10^4^ cells/well in 100 µL standard growth media. Cells were treated with sFlt-1 recombinant protein (R&D systems) and 2% serum from recruited women, respectively, and incubated at 37°C and 5% CO_2_ for 24 h. Following treatments, assays were initiated by removing culture media from each well with a non-buffered DMEM media, to allow temperature and pH equilibrium. Subsequently, OCR and ECAR were measured simultaneously three times to establish a baseline rate for 3-5 min. Then, to evaluate mitochondrial function, we used well-characterized inhibitors of the mitochondrial function. This approach allowed us to evaluate the contribution of different parameters of the mitochondrial function.

Oligomycin (1mM) (Sigma Aldrich), carbonyl cyanide 4-(trifluoromethoxy)phenylhydrazone (FCCP) (0.5mM) (Sigma Aldrich) and a mixture of rotenone and antimycin A (Rot/AntA) (1mM) (Cayman Chemicals) were injected into each well sequentially, with intervals of 3-5 min of mixing between the injections, to respectively inhibit the ATP synthase, uncouple oxidative phosphorylation, and estimate non-mitochondrial respiration. This experiment measures six parameters of the mitochondrial function: basal oxygen consumption, ATP-linked oxygen consumption, proton leak, maximal oxygen consumption, reserve capacity, and non-mitochondrial oxygen consumption. A representation of the data obtain from this experiment is shown in ***Supplementary Figure 1 A***. After the completion of the determinations, OCR and ECAR measurements were normalized to protein content by the Bradford method.

Based on the determinations obtain in the experiments of mitochondrial function, we calculated the cellular respiratory control ratio and energy phenotype map.

Cellular respiratory control ratio (RCR) was assessed by measuring parameters of the mitochondrial function. RCR shows the coupling state of mitochondria, representing the ratio of oxidative phosphorylation (OXPHOS) capacity (state 3) divided by the rate of resting respiration (state 4). Uncoupled or maximal RCR (RCR max) was obtain as the ratio of FCCP-stimulated and oligomycin-inhibited mitochondrial respiratory rates. The absolute or basal RCR (RCR basal) was approximated as the ratio of basal and oligomicyn-inhibited (basal RCR) mitochondrial respiratory rates (Brand and Nicholls, 2011).

The energy phenotype map represents both mayor energetic pathways simultaneously, OXPHOS and glycolysis. Basal and maximal OCR are plotted in Y axis over the basal and maximal ECAR values in the X axis (***Supplementary Figure 1 B***). This representation let us to identify a larger view of the cellular metabolic state and the visualization of alterations in oxygen consumption and concomitant increase in the glycolytic response (metabolic switch).

### Aerobic glycolysis

To evaluate the glycolytic pathway, ECs and HTR-8/SVneo cells were seeded in V7 Seahorse micro-well plates at 3.5-4.0 × 10^4^ cells/well in 100 µL standard growth media. Cells were treated with sFlt-1 recombinant protein (R&D systems) and incubated at 37°C and 5% CO_2_ for 24 h. Following treatments, assays were initiated by removing culture media from each well with a non-buffered DMEM media without glucose, to allow temperature and pH equilibrium. Subsequently, OCR and ECAR were measured simultaneously three times to establish a baseline rate for 3-5 min. To evaluate glycolytic response, cells were subjected to glucose (5.5 mM) (Sigma Aldrich), oligomycin (1mM) (Sigma Aldrich) and 2-deoxi-glucose (2-DG) (100 mM) (Sigma Aldrich), subsequently, to respectively induce glycolysis, inhibit ATP synthase and estimate non-glycolytic acidification.

This approach identified the glycolytic rate and glycolytic reserve capacity of ECs exposed to sFlt-1 exogenous administration. A representation of the data obtain from this experiment is shown in ***Supplementary Figure 1 C***. After the completion of the determinations, OCR and ECAR measurements were normalized to protein content by the Bradford method.

# Supplementary Figures and Tables

## Supplementary Table

Supplementary Table 1: Clinical characteristics of the recruited women. Data is presented as means ± SEM. Primiparous variable values are presented in %. *P=<0.05, vs. non pregnant controls (CTL), #P<0.01 vs. NOR group. N.A. Data not available.

## Supplementary Figures

Supplementary Figure 1. Parameters of mitochondrial bioenergetics and cellular metabolism in intact cells. Oxygen consumption rates (OCR) determinations and parameters of mitochondrial function. (B) Energetic phenotype map shows the metabolic state of cells and (C) Extracellular acidification rates (ECAR) determination and parameters of the glycolytic function.

Supplementary Figure 2. sFlt-1 levels measured by ELISA. Serum levels were measure in non-pregnant controls (CTL), normotensive (NOR) and preeclamptic (PE) women by ELISA. Data is presented as means ± SEM. (n=6), *P<0.05 vs. CTL, #P<0.05 vs. NOR. ANOVA (Bonferroni’s pos hoc test).

Supplementary Figure 3. sFlt-1 induced mitochondrial bioenergetics dysfunction in vitro. (A) Ratios of basal and maximal (Max) respiratory control (RCR) (State 3/state 4) and (B) Extracellular acidification rates (ECAR) yield by time, demonstrate a metabolic phenotype switch from mitochondrial phosphorylation to glycolysis, in endothelial cells exposed to 0, 10, 25 and 50 ng/mL of exogenous sFlt-1 for 24 h. Data is presented as means ± SEM (n=5), *P<0.05, **P<0.01, ***P<0.001 vs. maximal RCR measured in untreated controls. ANOVA (Bonferroni’s pos hoc test).

Supplementary Figure 4. sFlt-1 acts as a mitochondrial bioenergetics disruptor. (A) Cell proliferation counts measured in endothelial cells and (B) trophoblasts cultured in glucose and galactose media and also exposed to 50 ng/mL of exogenous sFlt-1 for 24, 48 and 72 h. Data is presented as means ± SEM. (n=3), *P<0.05, vs. galactose exposed cells. #P<0.05, vs. glucose exposed cells. ANOVA (Bonferroni’s pos hoc test).
